# Artificial selection with traditional or genomic relationships: consequences in coancestry and genetic diversity

**DOI:** 10.3389/fgene.2015.00127

**Published:** 2015-04-07

**Authors:** Silvia Teresa Rodríguez-Ramilo, Luis Alberto García-Cortés, María Ángeles Rodríguez de Cara

**Affiliations:** ^1^Departamento de Mejora Genetica Animal, Instituto Nacional de Investigacion y Tecnologia Agraria y AlimentariaMadrid, Spain; ^2^Laboratoire d'Eco-anthropologie et Ethnobiologie, Museum National d'Histoire NaturelleParis, France

**Keywords:** genomic selection, coancestry, inbreeding, breeding value, genetic diversity

## Abstract

Estimated breeding values (EBVs) are traditionally obtained from pedigree information. However, EBVs from high-density genotypes can have higher accuracy than EBVs from pedigree information. At the same time, it has been shown that EBVs from genomic data lead to lower increases in inbreeding compared with traditional selection based on genealogies. Here we evaluate the performance with BLUP selection based on genealogical coancestry with three different genome-based coancestry estimates: (1) an estimate based on shared segments of homozygosity, (2) an approach based on SNP-by-SNP count corrected by allelic frequencies, and (3) the identity by state methodology. We evaluate the effect of different population sizes, different number of genomic markers, and several heritability values for a quantitative trait. The performance of the different measures of coancestry in BLUP is evaluated in the true breeding values after truncation selection and also in terms of coancestry and diversity maintained. Accordingly, cross-performances were also carried out, that is, how prediction based on genealogical records impacts the three other measures of coancestry and inbreeding, and viceversa. Our results show that the genetic gains are very similar for all four coancestries, but the genomic-based methods are superior to using genealogical coancestries in terms of maintaining diversity measured as observed heterozygosity. Furthermore, the measure of coancestry based on shared segments of the genome seems to provide slightly better results on some scenarios, and the increase in inbreeding and loss in diversity is only slightly larger than the other genomic selection methods in those scenarios. Our results shed light on genomic selection vs. traditional genealogical-based BLUP and make the case to manage the population variability using genomic information to preserve the future success of selection programmes.

## 1. Introduction

Best linear unbiased prediction (BLUP) is possibly the most common selection method in animal and plant breeding, where it is used to calculate estimated breeding values (EBVs). BLUP evaluations maximize the genetic gain given the data by increasing the accuracy of the predictions (Henderson, 1984). This method relies on both the additive relationship matrix between the individuals in the population, which are traditionally obtained from pedigree records, and on phenotypic records of the candidates to selection. Such is the power of BLUP that it is actually not only used in breeding programmes, but also in evolutionary ecology to estimate the strength of selection and evolutionary change (see Hadfield et al., 2010 for a review) and more recently in human genetics for the prediction of complex traits (Makowsky et al., 2011).

With the advent of high-throughput genotyping techniques and the development of chips containing thousands of single nucleotide polymorphisms (SNPs) at a reasonable cost, the implementation of genome-wide evaluations (Meuwissen et al., 2001; Goddard and Hayes, 2007) is routinely used in many breeding programs, and conventional BLUP selection based on pedigrees is now migrating to genomic selection.

Genome-based EBV (estimated breeding values based on high-density marker data across the genome) have generally yielded a higher accuracy than pedigree-based EBV (Meuwissen et al., 2001; Goddard, 2009; Hayes et al., 2009; Sonesson et al., 2012; Rodriguez-Ramilo et al., 2014). This is because genetic markers provide a more accurate relationship matrices than pedigree data (Goddard, 2009), which accounts for the expected genetic relationships. For example, while the genealogical relationship between two full-sibs is 0.5, using molecular markers like high-density SNP chips, a more accurate value can be obtained, thus showing that the true relationship deviates from 0.5 (Visscher et al., 2006) and varies among pairs of sibs, depending on the segregation of the parental chromosomes (Garcia-Cortes et al., 2013).

Genomic selection can therefore lead to high levels of accuracy at an early age and generation intervals can be shortened leading to faster genetic gains within a specific breeding program. Furthermore, genomic selection not only has increased the accuracy in the breeding values, but also the increase in inbreeding per generation is lower than that obtained with conventional pedigree-based BLUP selection (Daetwyler et al., 2007; Sonesson et al., 2012). However, both traditional and genomic selection increase the levels of both inbreeding and coancestry, thus decreasing the pool of genetic diversity. This has wide-ranging consequences, as it is clear that such variation is needed for selection but also to avoid leading the population into extinction (Frankham et al., 2002). A crucial issue thus is a thorough understanding of the measures of coancestry between individuals and how they are affected by the relationship matrix used in the selection process, i.e., pedigree or genomic-based coancestries.

Traditionally, genealogical measures from pedigree records were used to calculate coancestry. As molecular markers became commonly used, estimates of genealogical coancestry from these markers were developed (Weir et al., 2006). It is only with the high-density panels that replacing genealogical coancestry with marker-based coancestry has become accepted as leading to more accurate predictions (Meuwissen et al., 2001; Meuwissen, 2007; Solberg et al., 2008) and to maintain more diversity in conservation programmes (de Cara et al., 2011). However, while the increase in accuracy in the EBVs using different marker types and densities is well-understood (Solberg et al., 2008; Jannink, 2010), the effect of different measures of coancestries in genomic and traditional selection has not received as much attention (Sonesson et al., 2012; Bjelland et al., 2013; Luan et al., 2014). For instance, genomic selection to estimate marker effects and predict the breeding values from them exploits the linkage disequilibrium between the markers in the panel and the causal mutations or QTL (Habier et al., 2007; de los Campos et al., 2010). When selection is performed via BLUP based on genomic relationships, the genetic gain is superior based on these relationships as compared to BLUP based on pedigree based relationships (Villanueva et al., 2005; Meuwissen, 2007) when the number of candidates for selection is large (Bastiaansen et al., 2012; Sonesson et al., 2012). Furthermore, selection based on genomic relationships also leads to lower increases in inbreeding and maintains more diversity (Sonesson et al., 2012; Liu et al., 2014).

In this study we analyse the effect of BLUP selection with four measures of coancestry on the genetic gain and on the increase in coancestry and inbreeding. For this purpose, we carry out simulations with three different genome-based relationship matrices and the matrix of genealogical relationships when inferring breeding values using BLUP. The three genomic measures of coancestry were: (1) based on shared segments of homozygosity (Fisher, 1954; Stam, 1980; Gusev et al., 2009), (2) using identity by state, that is, marker-by-marker similarity (Eding and Meuwissen, 2001; Caballero and Toro, 2002) and (3) based on a marker-by-marker count corrected by allelic frequencies (VanRaden, 2008). We measured the performance of selection with BLUP based on these four coancestries by analysing the genetic gain as measured with the true breeding values (TBVs).

## 2. Materials and methods

### 2.1. Base population

A base population was simulated with an effective size of 1000 individuals (half males, half females) during 10,000 generations until an equilibrium in the average genome-wide heterozygosity was reached. Every individual had a genome of 10 chromosomes of 1M with 10,100 biallelic positions each. Initially, every position in the genome carried alleles 0 or 1 at random, so that the average initial heterozygosity was 0.5. The mutation rate per position and generation was 2.5×10^−3^. Every generation during the creation of the base population we firstly performed mutations in every individual, then chose a male and a female at random with replacement and produced an offspring with recombination. The number of recombinations per chromosome were sampled from a Poisson distribution and the recombination positions were drawn from a uniform distribution. The base populations were generated with a fortran 90 code available upon request.

### 2.2. Selection

We performed 100 replicates of each scenario here studied by selecting 1000 polymorphic positions from this base population to be later used as selective loci (also known as QTLs in the literature). We sampled these selective loci from positions with 0.05 < *p_j_* < 0.95, where *p_j_* is the allelic frequency of allele 1 at locus *j*. Note thus that the 100 replicates are all created from one single base population by selecting different selected loci and different individuals in each replicate.

Founder individuals for each replicate were chosen at random from the base population without replacement, by drawing an equal number *N* of founder sires and dams from the base population to create generation 0. We then performed 6 generations of random mating to record the genealogy.

From generation 7 onwards we performed truncation selection for 15 generations (up to generation 21), by selecting the best 50% of the sires and 50% of the dams according to each individual's expected breeding value. These sires and dams were mated at random to produce *N* sires and *N* dams for the next generation.

The default parameters used in our simulations are *N* = 50, a marker density of 10,100 markers per chromosome and a trait with heritability of *h*^2^ = 0.25. To have a thorough understanding of the dependence of the results on population size, heritability and marker density, we also studied the following scenarios: we evaluated population sizes *N* = 10 and *N* = 30, two other heritabilities of the quantitative trait (*h*^2^ = 0.10 and 0.50) and two other lower marker densities (2525 and 5050 markers per chromosome). Table 1 shows a summary of the simulated scenarios.

**Table 1:** **Parameters simulated for the different scenarios**.

***N***				***h*^2^**			**SNPs**		
	10	30	50	0.10	0.25	0.50	2525	5050	10,100
*N*	10	30	50	50	50	50	50	50	50
*h*^2^	0.25	0.25	0.25	0.10	0.25	0.50	0.25	0.25	0.25
SNPs	10,100	10,100	10,100	10,100	10,100	10,100	2525	5050	10,100

### 2.3. Calculation of phenotypic values and true and estimated breeding Values

We calculated the TBV of individual *i* as

(1) $$TBV_{i}=\sum_{j=1}^{n_{S}}a_{j}\left(x_{ij}-1\right),$$

where *x_ij_* is the number of copies of the allele 1 that individual *i* has at the *j*-th selective locus, *a*_*j*_ is the effect of the allele 1 at position *j* and *n*_*S*_ is the number of selective loci. The values of the effects *a* were drawn from a Gaussian distribution with mean zero and variance one. The phenotypic values (*y*_*i*_) of individuals were simulated as

(2) $$y_{i}=\mu +TBV_{i}+e_{i},$$

where *e_i_* is an error term for individual *i*, which was normally distributed with mean zero and variance σ^2^_*e*_. The phenotypic average μ was set arbitrarily to be equal to 100, although this value does not affect the EBV. The variance σ^2^_*a*_ was calculated as the empirical variance of the TBVs in the base population and σ^2^_*e*_ was adjusted so that the heritability was the desired *h*^2^. We had the phenotypic values for all individuals in the population.

EBV were calculated by solving Henderson's mixed model equations (Henderson, 1984) as follows:

(3) $$\left[\begin{array}{ll} X^{\prime }X & X^{\prime }Z\\ Z^{\prime }X & Z^{\prime }Z+\frac{\sigma_{e}^{2}}{\sigma_{a}^{2}}A^{-1} \end{array}\right]\left[\begin{array}{c} \hat{\mu }\\ E\hat{B}V \end{array}\right]=\left[\begin{array}{c} X^{\prime }y\\ Z^{\prime }y \end{array}\right],$$

where **X** and **Z** are the incidence matrices for the fixed and random effects, respectively and **A** is the relationship matrix. We assumed the variance components to be known. Equation (3) provides the pedigree-based breeding values, while genomic based breeding values can be obtained by replacing **A** and σ^2^_*a*_ in Equation (3) by the following genomic relationships and variances.

#### 2.3.1. Coancestry estimates

The four following genetic relationship matrices, here defined as twice the coancestry coefficient, were used:

1. *Additive relationship matrix* (**A**): This was calculated using the coancestry coefficient between individuals *i* and *k*, *f*_*A*_(*i, k*) following (Malecot, 1948) as the probability that two alleles taken at random, one for each individual, are identical by descent (IBD).
2. *Marker-by-marker relationship matrix* (**G**): In this case, the coancestry coefficient between individuals *i* and *k*, *f*_*G*_(*i, k*), is the probability that two alleles at a given locus taken at random from each individual are equal (identical by state, IBS). In this study, *f*_*G*_(*i, k*) was calculated as $f_{G}(i,k)=\frac{1}{4M}\sum_{n=1\;}^{M}\sum_{l_{i}=1}^{2}\sum_{m_{k}=1}^{2}I_{n}(l_{i},m_{k})$ where *M* is the number of markers and *I_n_*(*l_i_, m_k_*) is the identity of gamete *l* from individual *i* with gamete *m* from individual *k* at marker *n* and takes a value of 1 if both alleles are identical and 0 otherwise.
3. *ROH-based relationship matrix* (**R**): Following the study by de Cara et al. (2013), the coancestry coefficient based on shared segments of the genome between individuals *i* and *k* was calculated as $f_{R}(i,k)=\frac{1}{4L}\sum_{j}\sum_{a_{i}=1}^{2}\sum_{b_{k}=1}^{2}L_{j}(a_{i},b_{k})$, where *L_j_*(*a_i_, b_k_*) is the length of the *j*-th shared segment measured over the gametes *a_i_* and *b_k_* of individuals *i* and *k* and *L* is the length of the genome. For a region to be considered a shared segment, we used a minimum length of 100 shared contiguous markers. The idea behind this segment-based relationship is that a segment shared between parents is a potential run of homozygosity (ROH) in the offspring.
4. *Marker-by-marker corrected by allele frequencies relationship matrix* (**V**): Following VanRaden (2008) a measure of coancestry *f_V_*(*i, k*) between individuals *i* and *k* can be calculated as
  (4) $$f_{V}(i,k)=\frac{1}{M}\sum_{n=1}^{M}\frac{\left(g_{in}-p_{n}\right)\left(g_{kn}-p_{n}\right)}{p_{n}\left(1-p_{n}\right)},$$
  where *g_in_* refers to the gene frequency value genotypes 00, 01, and 11, coded as 1, 0.5, and 0, respectively, of individual *i* at locus *n*. Gene frequency is half the number of copies of the reference allele 1 and *p_n_* is set at 0.5 (Forni et al., 2011).

Every generation we estimated the additive variance of the base population using restricted maximum likelihood (REML). We performed REML by using a Monte Carlo expextation-maximization (EM) algorithm (Guo and Thompson, 1991) to avoid the repeated matrix inversion required by exact algorithms (Meyer, 1991). Additive variances were estimated after six thousand iterations and discarding the first 1000. As for the base population, the fortran 90 code for the selection process is available upon request.

## 3. Results

As summarized in Table 1, we studied a combination of three population sizes, three heritabilities of the trait and three marker densities. The default case unless otherwise stated is the case of 10,100 markers per chromosome, heritability *h*^2^ = 0.25 and a population size with 50 males and 50 females per generation.

### 3.1. Distribution of coancestries

**Figure 1:**
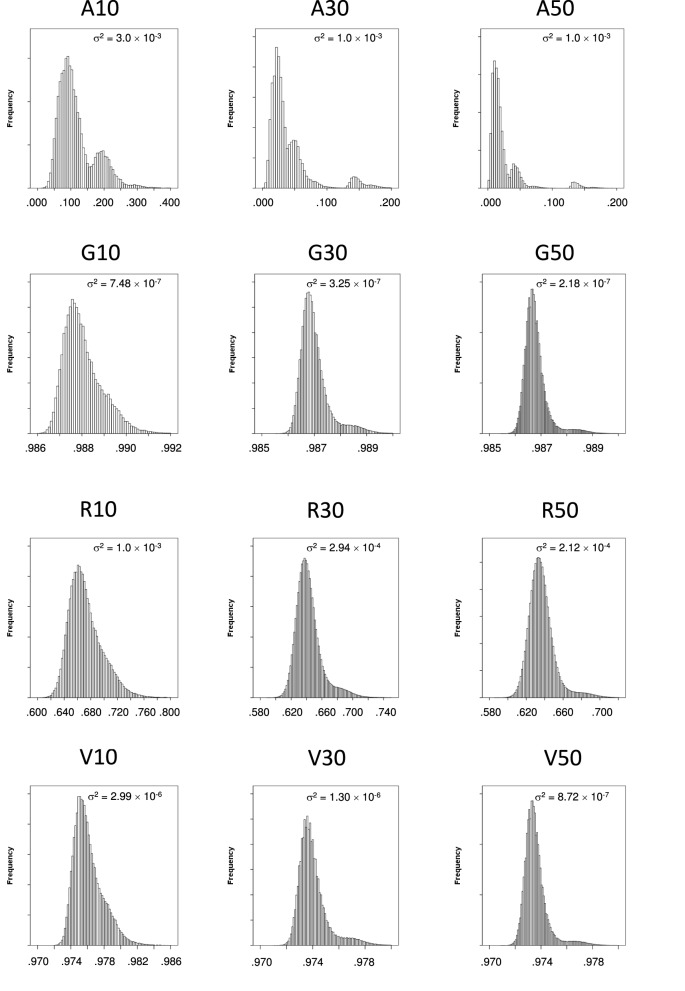
**Histograms of the coancestries at generation 6 right before selection**. Top row shows the histogram for genealogical coancestry *f_A_* for 10, 30, and 50 individuals from left to right. Similarly, the second row shows the histogram for molecular marker-by-marker coancestry *f_G_*. The third row shows the histograms for segment-based coancestry *f_R_*, for *N* = 10, *N* = 30, and *N* = 50 from left to right. The bottom row shows the histogram for molecular marker-by-marker coancestry corrected by allelic frequencies *f_V_*, for *N* = 10, *N* = 30, and *N* = 50 from left to right. The variance of each histogram is given within each plot.

Most likely, the differences in our results are going to be due to the distribution of coancestries, as the different selection strategies here performed are based on the matrix of relationships between individuals. We show in Figure 1 the distributions for the four measures of relationships prior to selection and give the variance within each figure. There we can see how the shape of the distribution of the genealogical coancestry is multimodal, given the sparse nature of the genealogical coancestry matrix and its distribution has the largest variance of all coancestry matrices, as well as the lowest mean. The distribution of coancestries *f_V_* and *f_G_* are fairly similar, the first one having a lower mean and a slightly larger variance although both distributions have a very small variance. Lastly, the distribution of coancestries *f_R_* has a mean considerably lower than the other genomic coancestries *f_V_* and *f_G_* and a substantially larger variance.

### 3.2. Genetic gain

**Figure 2:**
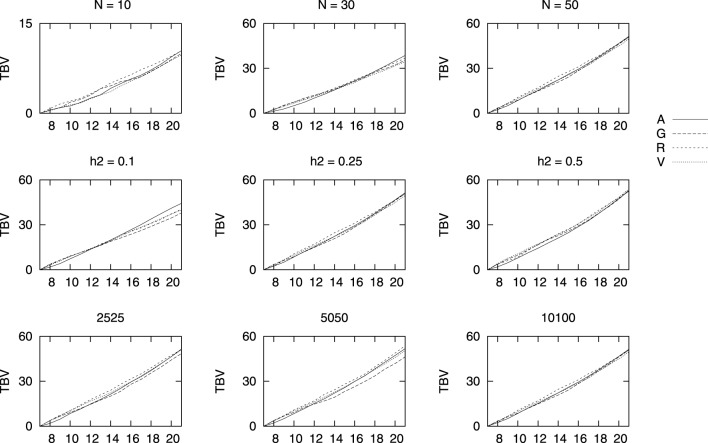
**Mean true breeding values (TBV) for different marker densities (bottom row), heritability (middle row), and population size (top row) vs. generations of selection**. TBV values are shown minus the value right before truncation selection started. The default values are 50 sires and 50 dams, a heritability of 0.25 and 10,100 markers, unless the value at the top of the figure indicates otherwise.

Changes in TBVs obtained with the four relationship matrices for three population sizes *N* = 10, *N* = 30, and *N* = 50, three heritabilities *h*^2^ = 0.1, *h*^2^ = 0.25, and *h*^2^ = 0.50, as well as three marker densities of 2525, 5050, and all 10,100 per chromosome are shown in Figure 2 vs. generations. We only show results after generation 7, when selection starts. For a better comparison between the different coancestries here used, we show the value at each generation minus the initial value right before selection (i.e., at generation 7). Overall, all four methods performed similarly in terms of genetic gain for the sizes here studied. As expected, the final TBV increased with the number of individuals and with the heritability of the trait. The density of markers had no effect when selecting with the genealogical coancestry *f_A_*, as expected, and, within the range of densities here studied no differences were detected in the genetic gains achieved by the genomic based estimates *f_V_* and *f_G_*. The most surprising result is that for a low density of markers, the genetic gain is larger performing selection based on *f_R_*. It must be noticed that the size for a region of homozygosity to be considered as such was kept constant and thus, a ROH of 100 contiguous markers covers a much longer stretch than for 10,100 marker per chromosome. This is also surprising as it has been pointed out that the longer the ROH, the more correlated ROH-based inbreeding is with genealogical inbreeding.

### 3.3. Changes in relatedness

**Figure 3:**
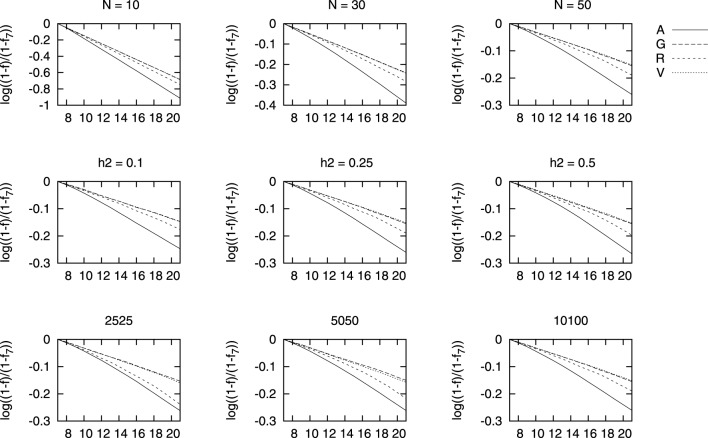
**Change in each coancestry for different marker densities (bottom row), heritability (middle row), and population size (top row) vs. generations of selection**. The change in each coancestry is shown as $log\left(\frac{1-f}{1-f_{7}}\right)$.

We show in Figure 3 results for the changes in each of the four measures used of coancestry with each selection scenario. We have used a logarithmic scale as overall, the differences between genealogical based selection and genomic based selection were very large. That is, line “A” shows the results for genealogical coancestry resulting from selecting based on this coancestry *f_A_* and so on for scenarios G, R, and V. The results for inbreeding are not shown as they display a very similar pattern. In order to better appreciate the differences between the four measures of coancestries, we show *log*[(1 − *f*)/(1 − *f*_7_)] in Figure 3. In this way, we compare the speed of increase in each average coancestry scaled with their values at generation 7 (*f*_7_), right before selection started. The increase in genealogical coancestry (the decay in this log scale) is the largest, followed by ROH-based coancestry. Changes in *f_V_* and *f_G_* are hardly distinguishable and very similar to *f_R_* for small heritability. The smaller the population, the larger the increase in any measure of coancestry. The differences in *f_G_* and *f_V_* are hardly different as heritability increases from *h*^2^ = 0.25 to *h*^2^ = 0.5.

**Figure 4:**
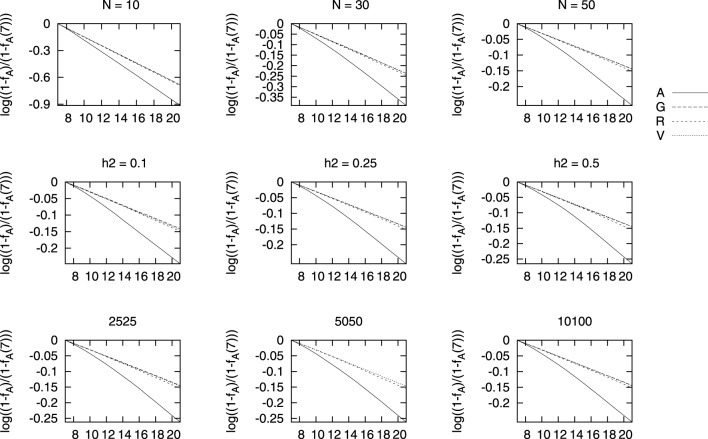
**Changes in genealogical coancestry for different marker densities (bottom row), heritability (middle row), and population size (top row) vs. generations of selection**. The change in each coancestry is shown as $log\left(\frac{1-f_{A}}{1-f_{A}(7)}\right)$, where *f_A_* is the genealogical coancestry at each generation and *f_A_*(7) is the value before selection starts.

In Figure 4 we show a similar plot for the change in pedigree based coancestry obtained under each selection scenario. All cases studied showed that the three genomic based selection led to lower increases in pedigree-based coancestry and the differences between the selection based on genomic relationships are hardly noticeable. The results are very similar for *f_V_* and *f_G_* based BLUPs on genealogical coancestry and it seems that *f_R_*-based BLUP leads to slightly larger genealogical coancestries.

### 3.4. Diversity maintained

**Figure 5:**
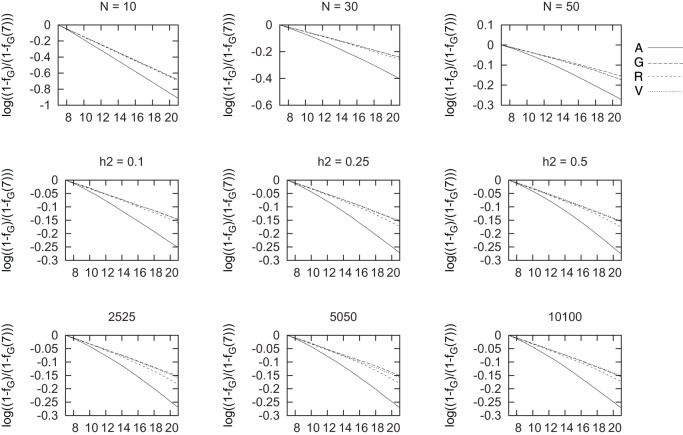
**Changes in molecular coancestry as a measure of change in diversity for different marker densities (bottom row), heritability (middle row), and population size (top row) vs. generations of selection**. This change is shown as $log\left(\frac{1-f_{G}}{1-f_{G}(7)}\right)$, where *f_G_* is the value of molecular coancestry at each generation and *f_G_*(7) is the value before selection starts.

As a measure of the diversity maintained we used *f_G_*, as this is directly related to observed heterozygosity. In Figure 5, we show the changes on this marker-by-marker relatedness over generations when selection was carried out using the four strategies analyzed. As previously done for all coancestries and for genealogical coancestry, we show its rate of decrease by plotting *log*(1 − *f_G_*) in Figure 5, minus this value right before starting selection *log*(1 − *f_G_*(7)) to compare all selection processes. Therefore, in this scale, the largest decrease means the largest increase in *f_G_*.

It is important to highlight that the highest loss in genetic diversity (the largest increase in *f_G_*) was observed for the selection based on the additive relationship matrix without exception. The fastest decay is for the smallest population size of *N* = 10 and then for *N* = 30 and this decay is largest with decreasing population size than heritabilities or marker densities. Within each scenario, it seems that initially most diversity is maintained selecting with the genomic coancestries and the difference between *f_G_* and *f_V_* is small. The difference between *f_G_* or *f_V_* and *f_R_* is small, though *f_R_*-based BLUP can lead to slightly larger decreases in molecular coancestry than the other two genomic measures of relatedness, especially for small marker density. That is, *f_R_*-based BLUP maintains slightly less genetic diversity than the other genomic based BLUPs.

## 4. Discussion

We have shown here results for truncation selection performed with four different measures of coancestry: *f_A_*, *f_G_*, *f_R_* and *f_V_*. All results shown are selecting the top 50% of sires and dams and we have compared results with three different population sizes, three different heritabilities of the selected trait and three different number of markers per chromosome.

We have performed 6 initial generations of random mating to have a deeper pedigree and have a fairer comparison between molecular markers which record the whole population history and genealogies, which are usually only stored when the selection programme starts.

There seems to be currently a consensus that genomic BLUP selection, whereby we mean selection based on genomic measures of relatedness, is superior to traditional pedigree-based BLUP selection (Daetwyler et al., 2007, 2010; Sonesson et al., 2012) in terms of higher genetic gain and lower increase in inbreeding. However, few studies have paid attention to the loss of genetic variability caused by each selection strategies of selection (Jannink, 2010; Bastiaansen et al., 2012; Heidaritabar et al., 2014; Liu et al., 2014). We discuss our main conclusions and the differences with these previous studies below.

### 4.1. On genetic gain

One of the main properties of BLUP is that by definition, the largest gain is obtained when the additive genetic variance of the base population is known. This is a difficult task, as for a large number of loci under selection which may be linked, the standard formula of $\sigma_{a}^{2}=\sum_{j=1}^{n_{S}}2p_{j}\left(1-p_{j}\right)a_{j}^{2}$ (Falconer and Mackay, 1996) does not apply. Furthermore, this variance is not appropriate when the performed BLUP relies on the genomic relationships *f_G_*, *f_R_* or *f_V_*. Thus, we estimated the additive variance components using REML. While it is well-known that the estimates obtained with REML are more accurate for larger population sizes than the ones here studied, the differences between the four selection strategies here studied are small. We think that these differences are independent of whether the variance could have been better estimated. We believe that a more accurate estimate of the variance of the base population would lead to larger gains for all four BLUPs here performed and the differences in the trends would stay the same.

Overall, the genetic gain was very similar with the four relationship matrices, although BLUP based on *f_R_* performed slightly better than the other BLUPs in terms of gain for lower marker densities. It also performed somewhat better for small population size and the intermediate heritability here studied, at least up to generation 18 (i.e., after 10 generations of selection). It is worth emphasizing that for the lower marker densities here studied, we kept the same threshold size of 100 consecutive markers for a ROH to be considered as such. That means that for 2525 markers per chromosome, such ROH would cover a section of about 4 cM, while for 10,100 a ROH of 100 consecutive markers covers 1 cM. Thus, for higher marker densities, it is likely that the gains could be increased by using a larger threshold for what is considered a ROH.

As expected, the final TBVs were larger for larger population size and for higher trait heritability. This is due to the larger genetic variance for larger population sizes in which selection can act upon, while the negative effects of inbreeding are reduced with higher population sizes. It is however somewhat surprising that the differences are small in genetic gain with marker densities for the genomic relationships matrices, particularly for *f_V_* and *f_G_*. This could indicate that a density of 2525 markers per chromosome would give the same correlation between the true genomic relationship if we had the whole sequence and that estimated with such marker density (Rolf et al., 2010).

It is likely that the lack of differences in genetic gain between the genomic and pedigree based relationships stems from the fact that we use the marker data to infer the relationships, but not to estimate the marker effects. It is in this later scenario where genomic selection seems considerably superior to traditional pedigree BLUP, although it depends on having enough training generations where both phenotypes and genotypes are recorded, as reviewed recently by Van Eenennaam et al. (2014).

In genomic selection, markers that densely cover the genome are expected to be in complete or partial linkage disequilibrium with the trait under selection. Genomic prediction based on IBS information uses the family structure of the population (Habier et al., 2007), since the markers capture the linkage disequilibrium that arises from the family structure. Recently, Luan et al. (2014) have proposed an approach to predict genomic estimated breeding values from runs of homozygosity. This study indicates that runs of homozygosity yield a multi-locus measure of linkage disequilibrium and thus can account for larger chromosomal distances to capture linkage disequilibrium than genomic prediction based on IBS information. It is worth noting that in their study, Luan et al. (2014) used a somewhat different definition of segment that we have used here. They obtained slightly better predictions for the ROH-based scenarios than for other genomic-based scenarios. Our results seem to be in line with those obtained by Luan et al. (2014), although a more thorough analysis of both methods is required for a better comparison. The measure of ROH used by Luan et al. (2014) does not seem to require a threshold size for a run of homozygosity, but it requires knowledge of the mutation rates and the effective population size.

No significant differences were detected between the genetic gain obtained with *f_G_* and *f_V_*. The reason is that with the *f_G_* approach alleles that are IBD and IBS can not be distinguished and are both included in the coancestry (and inbreeding) measures. To express both pedigree- and genomic-based estimates in the same scale several methodologies have been proposed Toro et al. (2011). However, these methods are generally inaccurate and their performances are very similar to those for *f_G_* Toro et al. (2002).

Sonesson et al. (2012) compared breeding schemes by simulating truncation or optimum contribution selection. They estimated breeding breeding values based on genome- or pedigree-based BLUP and recorded trait information on full-sibs of the candidates. This study concluded that to control inbreeding it is necessary to account for it on the same basis as what is used to estimate breeding values. Our results are in general agreement to those of Sonesson et al. (2012) regarding the genetic gain both with genomic- and pedigree-based selection procedures and with those of Bastiaansen et al. (2012), where higher accuracies were obtained for the genomic methods than for traditional pedigree-based BLUP.

### 4.2. On coancestries and inbreeding

As we have shown in Figure 4, the largest increases in coancestries, and similarly for inbreeding, is for the genealogical coancestry compared to other genomic measures of coancestry. At the same time, this increase in genealogical coancestry is larger with traditional pedigree-based BLUP than for any other BLUP here performed. This is in line with what Sonesson et al. (2012) obtained using BLUP combined with optimal contributions to control the increase in inbreeding, that the rate of increase in pedigree coancestry is higher for the pedigree-based selection scenario than for the genome-based selection approaches. This can be observed regardless the population size, the true heritability, or the density of markers. Bastiaansen et al. (2012) showed similar differences between traditional pedigree-based and genomic-based BLUP. They also showed how this difference built up with generations and was hardly noticeable after one round of selection. This study showed that the increase in inbreeding hardly depended on the genomic architecture of the selected trait, which is in line with what we observe in Figure 4, where the increase in coancestry seems independent of the marker density or the heritability of the trait. In agreement with Bastiaansen et al. (2012), we have also shown that genomic-based BLUPs can track Mendelian sampling within families, which is not possible with genealogical-based BLUP. Our results are apparently in contrast with the recent study of Liu et al. (2014), who obtained a lower increase in inbreeding for the larger heritability 0.25 in their study compared to that obtained for *h*^2^ = 0.05. This is most likely due to the fact that they looked at the results after 8 generations of selecting the top 25% candidates each generation, while we have performed selection on the top 50% candidates and looked at the increase of coancestry after 14 generations of selection. This shows the importance of understanding the dynamics at different generation intervals.

Liu et al. (2014) debated whether using genealogical records would be a good measure of inbreeding, as it reflects expected relationships and not the actual ones. They proposed measuring inbreeding then based on runs of homozygosity, and obtained that genomic-based BLUPs lead to lower increases on genealogical inbreeding as compared to phenotype BLUP, but this was not the case for inbreeding measured with ROHs. Our results for *f_R_* are very similar to those here presented for *f_A_*, and thus in the scenarios here studied, all genomic measures lead to lower increases in inbreeding whether we measure it with genealogies or with ROHs.

Our results show that the increase in genealogical coancestry seems slightly larger for ROH-based BLUP as compared to the other genomic-based BLUPs, although the differences are small.

### 4.3. On diversity maintained

It is well-known that selection reduces variation around the selected loci due to hitchhiking (Maynard-Smith and Haigh, 1974; Heidaritabar et al., 2014; Liu et al., 2014). Thus, if we aim at maintaining diversity while selecting favorables variants, it is important to understand which selection strategy works better overall. We evaluated *f_G_* as a measure of diversity maintained in the selection procedures simulated in the present study indicated that all genomic estimates maintained more variability than the pedigree-based ones. This result is in agreement with those also observed using simulated data but in the context of conservation programmes (de Cara et al., 2011), and with previous results in genomic selection (Liu et al., 2014).

An interesting study by Jannink (2010) showed that more variation could be maintained by placing more weight on favorable variants that are at low frequencies. This can potentially maintain more diversity both on the selected loci and on neutral loci. According to that study, this strategy leads to larger gains in the long-term, and thus this strategy could be optimal depending on how long is the long-term. Based on this study, it would be worthwhile studying whether placing weight on rare haplotypes could lead to a compromise between genetic gains and diversity maintained.

The study by Heidaritabar et al. (2014) has shown that changes in allelic frequencies are more localized around the selected loci with genomic based BLUP, while pedigree based BLUP leads to similar changes throughout the genome. Thus, it seems that genomic selection can lead to quick losses in genetic variation in specific regions of the genome, and thus great care is required if these regions provide potential adaptation of the breed.

In agreement with Liu et al. (2014), we have obtained that a larger heritability leads to larger decreases in diversity maintained when selecting with traditional BLUP. Similarly to what happened with genealogical inbreeding, the loss of diversity does not seem to depend on heritability when selecting with genomic-based BLUPs.

Interestingly, ROH-based BLUP seems to lead to slightly larger losses in diversity than the other genomic BLUPs, but massively smaller than pedigree BLUP. Consequently, a deep study of the factors involved in the definition of a ROH could help to improve the genetic gain obtained with this estimator while also keeping the a very high genetic variability.

In conclusion, in this study conventional pedigree based selection, which has been used for decades, results in similar genetic gains and does not maintain as much genetic variability as the genomic based selection methods. These results highlight the utility of genomic selection and also the need to manage the population variability using genomic information to preserve the future success of selection programs.

## Author contributions

All authors designed the study, performed the simulations and wrote the manuscript.

### Conflict of interest statement

The authors declare that the research was conducted in the absence of any commercial or financial relationships that could be construed as a potential conflict of interest.
